# Pleural plaques in lung cancer screening by low-dose computed tomography: prevalence, association with lung cancer and mortality

**DOI:** 10.1186/s12890-017-0506-3

**Published:** 2017-11-25

**Authors:** Mario Silva, Nicola Sverzellati, Davide Colombi, Gianluca Milanese, Carlo La Vecchia, Carlotta Galeone, Alfonso Marchianò, Ugo Pastorino

**Affiliations:** 1 Department of Medicine and Surgery (DiMeC), Section of Radiology, Unit of Surgical Sciences, University of Parma, Padiglione Barbieri, Via Gramsci 14, 43126 Parma, Italy; 2 Department of Thoracic Surgery, Fondazione IRCCS Istituto Nazionale dei Tumori, Milan, Italy; 3 Department of Clinical Sciences and Community Health, University of Milan, Milan, Italy; 4 Department of Radiology and Radiotherapy, Fondazione IRCCS Istituto Nazionale dei Tumori, Milan, Italy

**Keywords:** Lung cancer screening, Pleural abnormalities, Asbestos exposure, Pleural plaques, Self-disclosure of asbestos exposure, Post-test refinement of individual risk

## Abstract

**Background:**

To report the prevalence of pleural plaques in a lung cancer screening trial by low-dose computed tomography (LDCT) and to test the association with incidence of lung cancer and mortality.

**Methods:**

The LDCT of 2303 screenees were retrospectively reviewed with the specific aim of describing the prevalence and features of pleural plaques. Self-administered questionnaire was used to assess asbestos exposure. Frequency of lung cancer, lung cancer mortality, and overall mortality were detailed according to presence of pleural findings. Statistical analyses included comparison of mean or median, contingency tables, and Cox model for calculation of hazard ratio (HR) and its 95% confidence interval (CI).

**Results:**

Among male screenees, 31/1570 (2%) showed pleural abnormalities, 128/1570 (8.2%) disclosed asbestos exposure, 23/31 (74.2%) subjects with pleural plaques consistently denied exposure to asbestos. There was a trend for higher frequency of lung cancer among subjects with pleural plaques (9.7% vs 4.2%). Lung cancer in subjects with pleural plaques was always diagnosed in advanced stage. Subjects with pleural plaques showed HR 5.48 (95% CI 1.61–18.70) for mortality from lung cancer.

**Conclusions:**

Pleural plaques are a risk factor for lung cancer mortality that can be detected in lung cancer screening by LDCT, also in subjects that are not aware of asbestos exposure.

**Trial registration:**

NCT02837809 - Retrospectively registered July 1, 2016 - Enrolment of first participant September 2005.

## Background

Lung cancer screening by low-dose computed tomography (LDCT) has the main purpose of reducing lung cancer mortality in subjects that have several risk factors contributing to increased risk of lung cancer [1]. Asbestos exposure is a well-known risk factor for thoracic malignancies and non-malignant respiratory diseases [2–5], hence, the majority of lung cancer risk models include exposure to asbestos for pre-test calculation of lung cancer risk [3, 6, 7]. However, there is limited reliability on reference methods for the assessment of life-time exposure from occupational and, notably, non-occupational inhalation.

Signs of asbestos exposure can be detected by LDCT, with variable degrees of accuracy. Pleural plaques are among the chest abnormalities significantly related with exposure to asbestos [8, 9]. In the setting of professional exposure to asbestos, several studies reported the association between reduced survival and asbestos-related LDCT signs, including pleural plaques [4, 10]. The detection of pleural plaques and their prognostic value in non-occupational setting has not been investigated, yet. Lung cancer screening trials by LDCT provide the unique opportunity of investigating pleural plaques out of the specific setting of professional surveillance.

The aim of this study was to describe the prevalence of pleural plaques in a population of lung cancer screening participants, and to test their relation with incidence of lung cancer and mortality.

## Methods

The Institutional Review Board approved the Multicenter Italian Lung Detection (MILD) protocol, and written informed consent was obtained from all participants. Informed consent included retrospective evaluation of MILD data, as performed in this study. Therefore, specific consent was waived for this study. Eligibility criteria were as follows: age 50–75 years, current or former (having quit <10 years before enrollment) smokers with a tobacco burden ≥20 pack-years, and no history of cancer in the 5 years before enrollment. Details on screening strategy and lung cancer specific mortality rates of the MILD trial are published elsewhere [11]. In brief, the MILD trial enrolled a total of 4099 participants from September 2005 to January 2011. These subjects were randomly assigned to two groups: the early detection group (2376 participants) received a LDCT either every 12 months (1190 participants) or every 24 months (1186 participants), and the control group (1723 participants) received only primary prevention program with pulmonary function test and blood sample collection.

This investigation aimed to evaluate pleural abnormalities on baseline LDCT, and was thus based on the early detection group only. Baseline LDCT was available for 2303 out of 2376 participants (1570 men and 733 women; median age 60 years, interquartile range - IQR - 55 to 65 years). These subjects were evaluated for pleural abnormalities. The MILD protocol included self-administered questionnaire about medical history (i.e. oncologic history, cardio-vascular and respiratory anamnesis, metabolic diseases, and family history of lung cancer), smoking history (i.e. cigarette smokers were differentiated from other smokers, duration of smoking, amount of daily cigarette, pack-years, and Fagerstrom test) [12], exposure to asbestos, respiratory symptoms, subjective awareness of lung cancer risk related to smoking, and willing to quitting smoking [13]. Also, height and weight were recorded and body mass index (BMI) was calculated.

### Visual assessment of pleural plaques on LDCT

#### LDCT technique

LDCT was performed with a 16-detector CT scanner (Somatom Sensation; Siemens Healthcare, Forchheim, Germany) without contrast agent, and with standardized low dose acquisition protocol: potential 120 kVp, current 30 effective mAs, collimation 0.75 mm, rotation time 0.5 s, and pitch 1.5. For each LDCT examination, the reconstruction protocol was as follows: slice thickness 1 mm, slice increment 1 mm, and kernel b50. For this study, the reconstructed images were stored to a dedicated workstation for diagnostic work-up (Osirix, Pixmeo SARL, Bernex, Switzerland); window width (WW) and window level (WL) were optimized for assessment of pleura (soft tissue setting: WW 350 HU and WL 50 HU) and lung parenchyma (lung setting: WW 1600 and WL -600 HU).

#### Pleural findings

**Fig. 1:**
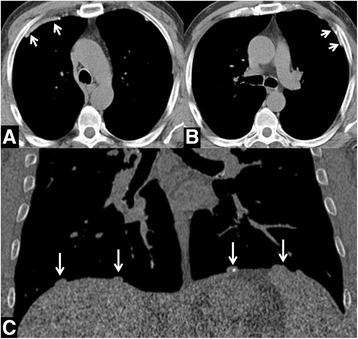
**a**-**c** Pleural plaques. **a**-**b** - Transverse CT image of the chest shows smooth solid thickening of pleural surface with clear-cut edge. **c** – Coronal reconstruction of the chest shows smooth solid thickening of pleural surface above the diaphragm, with scant calcification

LDCTs were reviewed by two radiologists for the detection of pleural abnormalities (both radiologists had three-year experience in lung cancer screening LDCT: 1201 LDCTs read by MS and 1102 LDCTs read by DC). A senior radiologist (NS, with 10-year experience in lung cancer screening LDCT) reviewed LDCT of all subjects with pleural abnormalities and selected those evocative of asbestos exposure, as described thereafter. Pleural plaques and diffuse pleural thickening were deemed findings evocative of asbestos exposure [8, 9]. In particular, pleural plaques were described as solid thickening of pleural surface with clear-cut edges, with or without calcification [14, 15] (Fig. 1). Diffuse pleural thickening was described as single continuous pleural thickening thicker than 3 mm, larger than 5 cm on axial plane, and longer than 8 cm in z-axis [8, 14]. From now on, all pleural abnormalities evocative of asbestos exposure will be referred as pleural plaques. All pleural plaques were visually scored according to a pre-formatted score sheet, adapted from the literature [14–17], namely: *a) distribution* according to side (unilateral or bilateral) and pleural anatomical compartment (subcostal, para-mediastinal, diaphragmatic, and fissural); *b)* presence of *calcifications*; *c) aspect* was described as smooth or nodular; *d) cumulative extent* was classified into 4 groups: smaller than 1 cm, between 1 cm and 25% of pleural surface, 25%–50% of pleural surface, and > 50% of pleural surface; *e) thickness* was classified into 4 groups: < 2 mm, 2–5 mm, 5–10 mm, and > 10 mm.

### Self-reported asbestos exposure

The self-administered questionnaire included disclosure of asbestos exposure. It was administered at the recruitment appointment and at any following LDCT appointment. Subjects with consistent positive disclosure about asbestos exposure over the consecutive questionnaires were deemed *exposed*. Conversely, *non-exposed* subjects were those with consistent negative disclosure or inconsistent disclosures along the consecutive questionnaires (e.g. positive disclosure at baseline questionnaire and negative disclosure at follow-up, or vice versa). The self-reported asbestos exposure was compared with the presence of pleural plaques to investigate awareness of exposure.

### Frequency of lung cancer and assessment of cause of mortality

Lung cancers were recorded from the surgical registry, in particular, histology and stage were reported [18]. Mortality from lung cancer and mortality from any cause were retrieved from the National Registry Office database. Data were updated to April 2016.

### Statistical analysis

Statistical analysis was selectively performed on male population to avoid multivariate model bias from gender-related variables (e.g. height, weight, and BMI) related to the odd distribution of pleural plaques between genders. The continuous variables were given as median with their interquartile range (IQR) and were analyzed using a two-sided Student’s t-test, if data were normally distributed (based on the Shapiro-Wilk statistic), or a two-sided Wilcoxon rank-sum test, if otherwise. The categorical variables were given as numbers and percentages and were analyzed using the contingency table analysis with the Chi-square or Fisher’s test, as appropriate.

The Cox model adjusted for age, BMI, pack-years, and self-disclosure of asbestos exposure was used to calculate the hazard ratio (HR) and the corresponding 95% confidence intervals (CI) for lung cancer incidence, lung cancer mortality, mortality from causes different from lung cancer, and overall mortality. All tests were two-sided and a *p*-value < 0.05 was considered as statistically significant. All statistical analyses were performed with MedCalc 12 (MedCalc Software, Belgium).

## Results

### Visual assessment of pleural plaques on LDCT

**Fig. 2:**
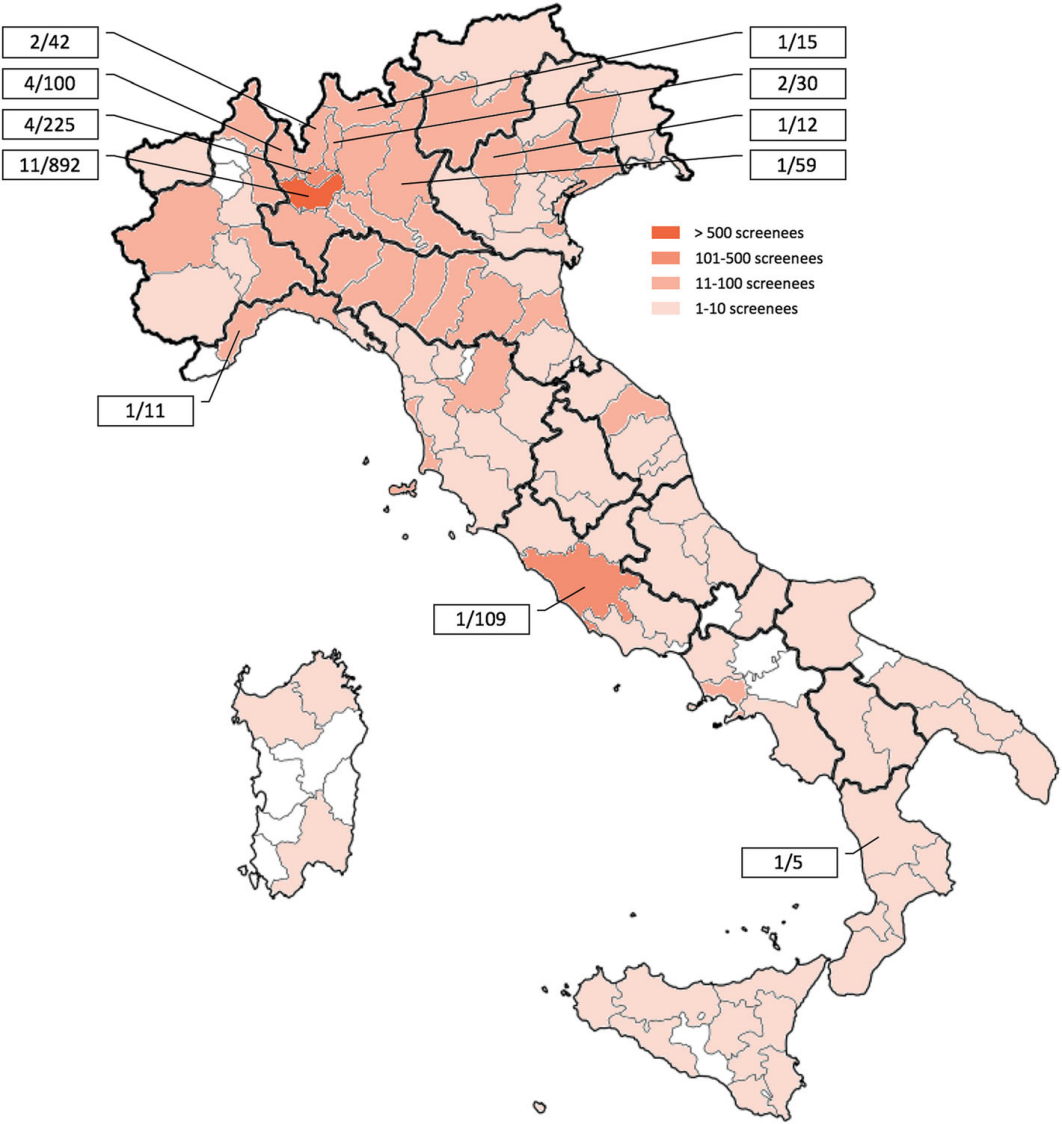
Geographic area of provenance of screenees are summarized according to numeric categories: 1–10 screenees, 11–100 screenees, 101–500 screenees, and > 500 screenees. The geographic distribution of pleural plaques is reported as the ratio between the number of screenees with pleural plaques and specific number of screenees from each geographic area (adapted from https://commons.m.wikimedia.org)

Pleural plaques were reported in 33/2303 subjects (1.4%), in particular 31/1570 men (2.0%) and 2/733 women (0.3%) (*p* < 0.001). Demographics, biometrics, smoking history, and self-disclosure of asbestos exposure of male screenees were reported in Table 1, according to presence of pleural plaques. Men with pleural plaques were older (*p* = 0.005), with similar biometrics and smoking history compared to men without pleural plaques. The geographic area of screenee provenance was summarized in Fig. 2 and the distribution of pleural plaques was detailed as the ratio of screenees from each area of provenance. LDCT features of pleural plaques were summarized in Table 2, notably pleural effusion was reported in 1/31 men (3.2%) and it was not associated with lung cancer or mesothelioma.

**Table 1:** Demographics, biometrics, smoking history, and self-disclosure of asbestos exposure in the overall male population and according to the presence of pleural plaques

	All men (*n* = 1570)	Men with pleural plaques (*n* = 31)	Men without pleural plaques (*n* = 1539)	*p* ^a^
Age [years]	58 [54–62]	62 [57–66]	58 [54–62]	0.0054
Height [cm]	174 [170–178]	177 [170–180]	174 [170–178]	0.2536
Weight [kg]	80 [73–88]	83 [75–92]	80 [73–88]	0.101
BMI [kg/m^2]	26.4 [24.4–28.7]	27.2 [24.8–30.2]	26.4 [24.4–28.7]	0.1949
Smoking history				
Current smoker	1007 (64.1%)	19 (61.3%)	988 (64.2%)	0.7103^b^
Former smoker	563 (35.9%)	12 (38.7%)	551(35.8%)	
Duration [years]	39 [34–43]	40 [36–45]	39 [34–43]	0.2501
Pack years	40.0 [32.0–54.0]	41.0 [35.0–61.5]	40.0 [32.0–54.0]	0.6378
Self-disclosure of asbestos exposure	128 (8.2%)	8 (25.8%)	120 (7.8%)	0.0025^b^

^a^Wilcoxon test

^b^Fisher exact test

**Table 2:** LDCT features of pleural plaques detected by LDCT in 31/1570 men

LDCT features of pleural plaques	*N (%)* 31 (100%)
Distribution by side:	
Bilateral	28 (90%)
Unilateral	3 (10%)
Distribution by anatomical compartment:	
More than one pleural compartment	21 (68%)
Subcostal only	9 (29%)
Para-mediastinal only	1 (3%)
Extent:	
< 1 cm	1 (3%)
1 cm to 25%	25 (81%)
25 to 50%	4 (13%)
> 50%	1 (3%)
Thickness:	
< 2 mm	1 (3%)
2–5 mm	7 (23%)
5–10 mm	19 (61%)
> 10 mm	4 (13%)
Calcification:	
Calcified	21 (68%)
Non-calcified	10 (32%)
Aspect morphology:	
Nodular aspect	19 (61%)
Smooth aspect	12 (39%)
Pleural effusion	1 (3%)

### Self-reported asbestos exposure

Asbestos exposure was consistently self-reported by 128/1570 men (8.2%), whereas 68/1570 (4.3%) provided inconsistent disclosure, and 1374/1570 (87.5%) consistently denied asbestos exposure. The consistent self-reported exposure to asbestos was significantly related to presence of pleural plaques (*p* = 0.003; Table 1), however the majority of men with pleural plaques consistently denied exposure to asbestos (23/31, 74.2%).

### Frequency of lung cancer and cause of mortality

Sixty-eight lung cancers were diagnosed in 1570 men over 14,533.3 person-years (467/100,000 person-years), until April 2016 (Table 3). In particular, the frequency of lung cancer was 3/31 in men with pleural plaques (1079/100,000 person-years) and 65/1539 in men without pleural plaques (456/100,000 person-years), with a non-statistically significant difference between the groups (*p* = 0.148). In particular, all men with pleural plaques showed stage 4 lung cancer. The mortality from lung cancer in men with pleural plaques was statistically higher compared to those without pleural plaques (*p* = 0.015), whereas the mortality from causes other than lung cancer was similar between the two groups.

**Table 3:** Comparison of lung cancer frequency, stage, and cause of mortality according to presence pleural plaques. The table includes events from the entire period of lung cancer screening (median follow-up 9.5 years). Notably, pleural plaques were searched on baseline LDCT, therefore detection of pleural plaques either preceded (e.g. event at incidence rounds) or was synchronous (e.g. event at baseline round) to each detailed event

	All patients (*n* = 1570)	Men with pleural plaques (*n* = 31)	Men without pleural plaques (*n* = 1539)	*p* ^a^
Total person-years follow-up	14,533.3	278.0	14,255.3	
Median person-years follow-up	9.5	9.4	9.5	
Patients with lung cancer	68 (4.3%)	3 (9.7%)	65 (4.2%)	0.148
Lung cancer stage				
I or II	39 (57.4%)	0	39 (60%)	0.073
III or IV	29 (42.6%)	3 (100%)	26 (40.0)	
Total deaths	87 (5.5%)	4 (12.9%)	83 (5.4%)	0.088
Lung cancer deaths	27 (1.7%)	3 (9.7%)	24 (1.6%)	0.015
Deaths from causes other than lung cancer	60 (3.8%)	1 (3.2%)	59 (3.8%)	1.0

^a^Fisher exact test

A single mesothelioma was diagnosed in a subject who did not have pleural plaques nor disclosed asbestos exposure.

Table 4 details the HR for overall or specific mortality and for incidence of lung cancer in subjects with pleural plaques. The HR for overall mortality was higher in men with pleural plaques (HR 2.02), allegedly conditioned by the exceptionally high HR for mortality from lung cancer (HR 5.48). Of note, the HR for lung cancer incidence was slightly but not significantly higher in men with pleural plaques (HR 1.84).

**Table 4:** Hazard ratios (HR)^a^ of overall mortality, lung cancer mortality and lung cancer occurrence among 1570 men according to pleural findings

	Pleural findings
	HR^a^ (95% CI)
Overall mortality (*N* = 87)	2.02 (0.73–5.56)
Mortality from lung cancer (*N* = 27)	5.48 (1.61–18.70)
Mortality from other causes than lung cancer (*N* = 60)	0.70 (0.10–5.10)
Lung cancer incidence (*N* = 68)	1.84 (0.57–5.93)

^a^Estimated from Cox model adjusted for age, BMI, pack-years, self-disclosure of asbestos exposure

## Discussion

This study reports 2% prevalence of pleural plaques in male participants of a lung cancer screening trial. Self-disclosure of asbestos exposure was recorded in 8.2% of male screenees, however a significant proportion of subjects with pleural plaques were not aware of asbestos exposure. Male screenees with pleural plaques showed a trend for higher risk of lung cancer and significant increase of lung cancer mortality compared to male screenees without pleural plaques.

The relationship between asbestos exposure and lung cancer has been investigated in several studies based on occupational surveillance programs for asbestos workers [19–22]. Current or recent occupational exposure to asbestos is a known risk factor for lung cancer with a reported two-fold increase of lung cancer risk among smokers [7]. Mastrangelo et al. reported progressive decrease of relative risk of lung cancer after 15–20 years since last exposure to asbestos [22], comparable to smoking cessation. Nevertheless, increased risk of thoracic malignancy has been demonstrated also for extremely low exposure, such as non-occupational and environmental exposure [23, 24]. Accordingly, several lung cancer risk models for selection of lung cancer screening population include asbestos exposure among risk factors, even in case of non-occupational exposure [3, 7]. Non-occupational exposure is likely to become more relevant in industrialized countries were asbestos ban was issued more than 20 years ago [10]. The assessment of non-occupational exposure to asbestos is extremely challenging, indeed more controversial that the assessment of occupational exposure [25]. Self-administered questionnaires have been employed to collect data on asbestos exposure in participants of lung cancer screening trials, despite this assessment demands specialized operators for thorough investigation of potential pollutant [26]. Our data showed that signs of asbestos exposure such pleural plaques were associated with increased risk of lung cancer mortality, including subjects who were not aware of occupational exposure. The increased risk was independent from tobacco burden. We encourage specific investigation of asbestos exposure (both occupational and non-occupational) for selection of subjects for lung cancer screening, even when tobacco burden does not meet the reference level for recruitment in such screening.

Previous lung cancer screening trials reported prevalence of asbestos-exposure ranging 5.6–7.3% [27, 28]. In our study, similar results were obtained by self-disclosure of asbestos exposure (overall self-disclosed exposure 6.5% - data not reported in results), however almost 75% of male screenees with pleural plaques did not disclose the exposure. This observation confirms that self-disclosure of asbestos exposure is not reliable, as it was reported in the literature [29]. Ledda et al. reported a significant increase of pleural plaques prevalence among subjects in an area with high environmental concentration of fibrous amphibole [30]. Of note, the CT features of pleural plaques reported by Ledda in environmentally exposed subjects were similar to those reported in our lung cancer screening population, in particular the majority of subjects showed plaque extent between 1 cm and 25% of pleural surface. Pleural plaques in subjects that are not aware of exposure to asbestos could be associated with non-occupational and environmental exposure, and this should be particularly emphasized in high-risk subjects that undergo lung cancer screening by LDCT.

Bach et al. showed increase of lung cancer risk in association to asbestos exposure in participants of lung cancer screening trials [31]. In our study, screenees with pleural plaques showed higher risk of lung cancer mortality compared to the remainder screenees. We underline that screenees with pleural plaques were diagnosed with advanced stage lung cancer. Hence, we suggest that increased mortality could have been driven by a more aggressive pattern of lung cancer in this group of screenees. Again, it is apparent that assessment of pleural plaques in lung cancer screening participant contributes to the detection of subjects with minor or unknown exposure. For this purpose, the objective assessment of pleural abnormalities could be regarded as potential marker of increased risk of lung cancer associated to asbestos exposure, and should be investigated for post-test refinement of subjective risk of lung cancer. Furthermore, from a demographic point of view, detection of pleural plaques in screenees that are not aware of the exposure should prompt active investigation of environmental exposure to asbestos in specific areas.

Among occupationally exposed subjects, the correlation between pleural plaques and lung cancer appears to be lower compared to the relative risk associated with asbestosis [10]. Both asbestosis and pleural plaques can be seen on LDCT, however pleural plaques are more specific than asbestosis findings in the absence of known exposure to asbestos. Vehmas et al. reported significant increase of lung cancer death in asbestos workers with pleural plaques [4]. This report in workers exposed to asbestos is confirmed by our data in the setting of lung cancer screening trial. Screenees with pleural plaques showed a trend for increased incidence of lung cancer, noteworthy the clinical evolution of lung cancer in these subjects was extremely aggressive. Lung cancer in subjects with pleural plaques were all diagnosed in stage 4 and, thus, lung cancer mortality was significantly higher compared to screenees with cancer diagnosis but without pleural plaques, suggesting that mortality from lung cancer in subjects with pleural plaques could be influenced by more factors rather than the simple incidence of lung cancer (e.g. aggressive biology). Risk models for post-test risk refinement are mostly based on the presence and characteristics of nodule and have been validated [32–34]. Looking forward to screening implementation, post-test risk model enrichment is fostered including other non-nodular LDCT findings such as emphysema and other interstitial lung abnormalities. Pleural plaques should be considered as additional non-nodular finding for post-test risk model.

This study has some limitations. Asbestos exposure was investigated by prospectively self-administered questionnaire, which is known to be inconsistent for assessment of asbestos exposure. However, self-administered questionnaire is the affordable option for implementation in lung cancer screening by LDCT. Moreover, asbestos exposure was not further investigated in subjects with pleural plaques and unaware of asbestos exposure. This could be regarded as a limitation because the specific description of exposure hazard in this subgroup could have provided information to improve anamnestic collection of this specific pollutant. Moreover, the asbestos exposure was investigated with a binary question (e.g. yes or no), without specific description of exposure duration and intensity. This limitation was driven by the management and financial plan within lung cancer screening trial with a large population of screenees. We foster future studies testing the correlation between detailed descriptors of asbestos exposure and lung cancer outcomes within lung cancer screening programs. Finally, parenchymal signs of asbestos exposure were not investigated, despite they are associated with risk of lung cancer. Notably, parenchymal signs were not scored because they are not specific for asbestos exposure when the exposure is not formally confirmed. It would be ideal that reliable information about asbestos exposure in lung cancer screening allow specific investigation of interstitial lung abnormalities related with the exposure, since they are significantly related with the risk of lung cancer [10].

## Conclusions

In conclusion, pleural plaques can be detected in a sizeable proportion of subjects undergoing lung cancer screening by LDCT. Screenees with pleural plaques show increased risk of lung cancer mortality, therefore pleural plaques could be considered in risk models for post-test refinement of subjective risk.

## Abbreviations

BMI: Body mass index
CI: Confidence interval
HR: Hazard ratio
IQR: Interquartile range
LDCT: Low-dose computed tomography
WL: Window level
WW: Window width
